# Factors Affecting Biochemical and Echocardiographic Indices in Type 2 Diabetes Mellitus Patients Without Overt Symptoms of Heart Failure: A Cross-Sectional Study

**DOI:** 10.7759/cureus.46904

**Published:** 2023-10-12

**Authors:** Raj Kishore Singh, Kunal Kumar Singh, Aradhana Singh, Imran Ahmed Khan, Subhash C Yadav

**Affiliations:** 1 Medicine, Baba Raghav Das Medical College, Gorakhpur, IND; 2 Cardiology, Baba Raghav Das Medical College, Gorakhpur, IND; 3 Obstetrics and Gynaecology, All India Institute of Medical Sciences, Gorakhpur, Gorakhpur, IND; 4 Community Medicine, Baba Raghav Das Medical College, Gorakhpur, IND; 5 Endocrinology, Sanjay Gandhi Postgraduate Institute of Medical Sciences, Lucknow, IND

**Keywords:** risk factor, prognosis, natriuretic peptide, heart failure, glycemic control, diabetes mellitus

## Abstract

Introduction

Diabetes is a known risk factor for heart failure (HF), and HF often manifests as a common cardiovascular event in people with type-2 diabetes mellitus (T2DM). Once HF is present, diabetes presents an especially adverse prognosis for subsequent morbidity and mortality. Brain natriuretic peptide (BNP) and n-terminal ProBNP (NT-proBNP) are used as diagnostic biomarkers for HF that are secreted by the ventricles in response to increased myocardial wall stress. If we could unmask some clinical and routine laboratory parameters affecting BNP and ejection fraction (EF), we can predict impending HF and take measures to prevent it. The current study was conducted to investigate the factors affecting BNP and EF for detecting potential HF in T2DM patients who do not exhibit overt HF symptoms.

Materials and methods

The present cross-sectional study was performed after obtaining ethical clearance from the Institutional Ethics Committee. T2DM patients consulting the Medicine Outpatient Department (OPD) of BRD Medical College Gorakhpur during a two-month period (from 20 July 2023 to 19 September 2023) with age >40 years and duration of T2DM >10 years. Multistage random sampling was done to recruit study participants, and 308 patients participated in the study. Informed consent was obtained from the recruited participants. The chi-square or Fisher's exact test (whichever was applicable) was used to explore the association between categorical variables. Correlation statistics were calculated using Spearman correlation among the NT-proBNP, EF, and other relevant variables. The Statistical Package for Social Sciences (SPSS) (version 21; IBM SPSS Statistics for Windows, Armonk, NY) was used for the analysis, and a two-sided p-value of < 0.05 was considered significant.

Results

Three hundred and eight diabetic patients satisfying inclusion and exclusion criteria were enrolled as study participants and completed the study. The mean age of the total study subjects was 60.82 ± 9.23 years. There were 161 (52.3%) male and 147 (47.7%) female participants, and about half (153/308, 49.7%) of the participants belonged to the age group 40-60 years. There was a statistically significant association (p = 0.01) between NT-proBNP and glycated hemoglobin. Statistically highly significant (p < 0.001) associations were found between NT-proBNP with duration of T2DM and EF. There was a strong negative correlation (correlation coefficient = -0.743) between EF and NT-proBNP, and this correlation was statistically highly significant with a p-value < 0.001.

Conclusion

Elevated NT-proBNP levels and impaired EF were found in a significant proportion of these patients, indicating an increased risk of cardiovascular complications. This study highlights a significant association between NT-proBNP and EF in patients with T2DM in those without overt heart failure symptoms. Furthermore, longer T2DM duration and higher HbA1c levels were found to be associated with elevated NT-proBNP levels, while longer T2DM duration and elevated NT-proBNP were linked to lower EF. These findings have important clinical implications, as they suggest that monitoring NT-proBNP levels in patients with T2DM without clinical features of overt heart failure may help identify those at risk for decreased EF and potentially prevent heart failure.

## Introduction

Type 2 diabetes mellitus (T2DM) is a heterogeneous group of disorders characterized by variable degrees of insulin resistance, impaired insulin secretion, and increased hepatic glucose production. The global burden of T2DM is on the rise, and it is estimated that diabetes will affect more than 430 million persons, 7.7% of the global adult population, by 2030 [1]. In patients with diabetes mellitus, advanced age, duration of the disease, insulin use, presence of coronary artery disease, and elevated serum creatinine are all independent risk factors for the development of heart failure (HF) [2]. HF is a clinical syndrome with an age-related increase in prevalence, from 1% at the age of 50-59 years to 10% at 75 years and older [3]. HF and T2DM confer a considerable burden on the healthcare system. T2DM can increase the risk of HF, whereas HF can accelerate complications of T2DM [4]. Diabetes is an established risk factor for HF, and HF often manifests as a common cardiovascular event in people with T2DM. Cardiovascular events may even present without typical chest pain [5]. The prevalence of unrecognized HF in those with T2DM is thought to be considerable. Generally, clinically manifesting HF is present in 10-30% of all subjects with T2DM, especially common at the age of 70 years and older, while 30-40% of all cases of acute or chronic HF have prevalent T2DM [6].

Brain natriuretic peptide (BNP) and N-terminal proBNP (NT-proBNP) are used as diagnostic biomarkers for HF that are secreted by the ventricles in response to increased myocardial wall stress. Current guidelines emphasize that patients suspected of HF with a BNP >100pg/mL or an NT-proBNP >125pg/mL must undergo echocardiography to confirm HF diagnosis and that patients with values below the cutoffs are very unlikely to have HF [7]. As potentiation of atherogenesis and cardiac dysfunction occurs in the presence of early diabetic symptoms and in the established disease, early implementation of strategies to manage risk factors and slow diabetes progression may help improve long-term outcomes. Such interventions may include effective treatments of hypertension and dyslipidemia, diet improvements, weight loss, and exercise as well as pharmacologic interventions aimed at newly identified therapeutic targets. If we could unmask some clinical and routine laboratory parameters affecting NT-proBNP and ejection fraction (EF), we can predict impending HF and take measures to prevent it. The current study was conducted to investigate the factors affecting NT-proBNP and EF for detecting potential heart failure in T2DM patients who do not exhibit overt heart failure symptoms.

## Materials and methods

Study design

The present cross-sectional study was performed after obtaining ethical clearance from the Institutional Ethics Committee of Baba Raghav Das (BRD) Medical College (approval number: 20/IHEC/2023). The confidentiality of the data was maintained.

T2DM patients consulting the Medicine Outpatient Department (OPD) of BRD Medical College during a two-month period (from 20th July 2023 to 19th September 2023) with age >40 years and duration of T2DM >10 years.

Sample size

The sample size was calculated based on a previous study using the following formula:

n = Z^2^ pq/d^2^ 

n = sample size

p = prevalence of HF among diabetics; q = 1-p

d = allowable error

z-statistic: The level of confidence of 95%, this is conventional. The z-score is 1.96

The prevalence of HF among diabetics was taken as 27.7% (based on an earlier study done by Boonman-de Winter et al.) [8]. Allowable error was taken as 5%.

N= (1.96)^2^ ×0.277× (1 - 0.277) / (0.05)^2^ = 308

Hence, the final sample size of this study comes to 308.

Inclusion and exclusion criteria

Patients aged ≥40 years of either sex, diagnosed with T2DM for more than a 10-year duration attending Medicine OPD, BRD Medical College, and willing to participate in the study were included in the study.

Patients not giving consent, with evidence of valvular disease, history of symptoms of peripheral artery disease, chronic obstructive pulmonary disease arrhythmias, and diagnosed with stage III/IV chronic kidney disease were excluded.

Operational definition

Diabetes was diagnosed by the American Diabetes Association Diagnostic Criteria for diabetes mellitus [9] as fasting plasma glucose (FPG) ≥126 mg/dL (7.0 mmol/L), two-hour plasma glucose ≥200 mg/dL (11.1 mmol/L) during an oral glucose tolerance test (OGTT), glycated hemoglobin (HbA1c) ≥6.5% (48 mmol/mol), or a patient with classic symptoms of hyperglycemia or hyperglycemic crisis with random plasma glucose ≥ 200 mg/dL (11.1 mmol/L). The diagnosis of HF is based on left ventricle ejection fraction (LVEF). The 2021 European Society of Cardiology (ESC) Guidelines [10] suggested three stages of HF along a spectrum. Left ventricular EF was estimated by Simpson's method and also by visual eyeball estimation, and it was performed by the same cardiologist for all participants. On the basis of echocardiography, every subject was classified as preserved ejection fraction (pEF), midrange ejection fraction (mrEF), and reduced ejection fraction (rEF) wherein pEF, LVEF ≥50%; mrEF, LVEF of 41-49%; and rEF (LVEF) ≤40% [11]. The NT-proBNP level up to 125 pg/ml was taken as normal and >125 pg/ml as elevated.

Sampling method

Multistage random sampling was adopted for recruiting study participants. In the first stage of sampling, three days per week were decided randomly by rolling a six-sided dice. This process was repeated until the data collection days for all eight weeks (from July 20, 2023, to September 19, 2023) of the data collection period were selected. By doing this, we prepared a date-wise list of 24 OPD days for data collection. The total duration of OPD days was divided into four quarters (08:00 a.m. to 10:00 a.m., 10:00 a.m. to 12:00 p.m., 12 p.m. to 02:00 p.m., and 2:00 p.m. to 4:00 p.m.) for the purpose of data collection. On every morning of the data collection day, a decision for data collection timing was made by the lottery method. Data collection was done during that selected two-hour duration of OPD. Twelve to fifteen diabetic patients per data collection day were recruited. The decision to include a patient in the study was made by flipping a coin. If heads were the result, the patient was asked to participate in the study; if tails was the result, they would be excluded. This procedure was done until the required sample size (308) was achieved. Written informed consent in the local language was taken from all the study subjects. Each study subject underwent evaluation for history, examination, investigations (including HbA1C and NT-proBNP level), and evaluation with echocardiography for ejection fraction as a routine part of their standard management protocol. All subjects were asked about their chief complaints, and questions were asked about dyspnea on exertion, exercise intolerance, fatigue, breathlessness, orthopnea, pedal edema, and any other chronic illness. A general examination was done to look for pallor, cyanosis, clubbing, edema, icterus, and lymphadenopathy. Every study subject’s blood pressure, pulse rate, BMI, and abdominal circumference were recorded. Every subject underwent detailed systemic examination, mainly cardiovascular and respiratory system examination with special emphasis on crepitation in the chest and raised JVP. A quick systemic review of other systems was done. As a routine part of management, a venous blood sample was taken in an EDTA vial and sent to the Central Pathology Lab, Nehru Chikitsalaya, BRD Medical College, Gorakhpur. In the Lab, it would be processed and analyzed in an automatic analyzer for HbA1C and other markers. For NT-proBNP analysis, an NT-proBNP Fast Test Kit (immunofluorescence assay; Getein Biotech, Inc., Nanjing, China) was used. Every participant’s echocardiography was obtained on a SonoSite M-Turbo medical ultrasound machine (Fujifilm SonoSite, Bothell, WA) with a 2.5-MHz transducer. We used two-dimensional measurement to calculate LVEF (left ventricular ejection fraction). The most often-used quantitative measure of systolic function is LVEF, which assesses how much of the left ventricular diastolic blood volume is evacuated during each cardiac cycle.

Data collection and statistical analysis

The data of participants were filled out in an Excel spreadsheet (Microsoft® Corp., Redmond, WA), and after data cleaning, it was imported into the Statistical Package for Social Sciences (SPSS) version 21 (IBM SPSS Statistics, Armonk, NY) for further analysis. Descriptive analysis was performed by applying appropriate statistical tests at the Department of Medicine in collaboration with the Department of Community Medicine of BRD Medical College, Gorakhpur. Categorical variables were presented in absolute numbers with percentage and continuous variables as mean and standard deviation (SD). The chi-square or Fisher's exact test (whichever was applicable) was used to explore the association between categorical variables. Normality was checked using the Kolmogorov-Smirnov test, and data were found to be not normally distributed. Correlation statistics were calculated using Spearman correlation between the NT-proBNP, EF, and other relevant variables. SPSS, version 21, was used for analysis, and a two-sided p-value of < 0.05 was considered significant.

## Results

In the present cross-sectional observational study, 350 patients attending Medicine OPD having T2DM with age >40 years and >10 years of T2DM duration were approached. Three hundred and eight diabetic patients satisfying inclusion and exclusion criteria were enrolled as study participants and completed the study. The mean age of the total study subjects was 60.82 ± 9.23 years.

There were 161 (52.3%) male and 147 (47.7%) female participants and about half (153/308, 49.7%) participants belonged to the age group 40-60 years. The clinical profiles of study participants are compiled in Table 1.

**Table 1:** Demographic and clinical profile of study participants (n = 308) SD, standard deviation; HbA1c, glycated hemoglobin; NT-proBNP, n-terminal pro-brain natriuretic peptide; pEF, preserved ejection fraction; mEF, mid-range ejection fraction; rEF, reduced ejection fraction

Study Participants	Category	Number	Percentage
Demographic Profile			
Age (Mean ± SD) 60.82 ± 9.23			
Age Group (Years)	40–60	153	49.7
	61–80	147	47.7
	80	8	2.6
Gender	Female	147	47.7
	Male	161	52.3
Clinical Profile			
Duration of Type-2 Diabetes (Years)	10–20	258	83.8
	21–30	46	14.9
	>30	04	1.3
HbA1c Level	<6.5%	29	9.4
	6.5–9.5%	228	74.0
	>9.5%	51	16.6
NT-proBNP Level (pg/dl)	Up to 125	210	68.2
	>125	98	31.8
Ejection Fraction	pEF (≥50%)	236	76.6
	mrEF (41–49%)	41	13.3
	rEF (≤40%)	31	10.1

There was a statistically significant association (p = 0.01) between age group, glycated hemoglobin, and NT-proBNP. Statistically highly significant (p < 0.001) associations were also found between NT-proBNP with a duration of T2DM and EF (Table 2).

**Table 2:** Association of NT-proBNP status with different variables of study participants p-value, significance value; HbA1c, glycated hemoglobin; NT-proBNP, n-terminal pro-brain natriuretic peptide; pEF, preserved ejection fraction; mEF, midrange ejection fraction; rEF, reduced ejection fraction

Variables of Study Participants		NT-proBNP Status			P-value
		Normal n = 210 (68.2%)	Elevated n = 98 (31.8%)	Total = 308	
Age Group (Years)	40–60	113 (73.9)	40 (26.1)	153	0.001
	61–80	96 (65.3)	51 (34)	147	
	≥80	1 (12.5)	4 (44.7)	8	
Duration of Type-2 Diabetes (Years)	10–20	208 (80.6)	50 (19.4)	258	< 0.001
	20–30	2 (4.3)	44 (95.7)	46	
	>30	0	4 (100)	4	
HbA1c	<6.5	23 (79.3)	6 (20.7)	29	0.004
	6.5–9.5	162 (71.1)	66 (28.9)	228	
	>9.5	25 (49.0)	26 (51.0)	51	
Ejection Fraction	pEF (≥ 50%)	201 (85.2)	35 (14.8)	236	< 0.001
	mEF (41–49%)	6 (14.6)	28 (90.3)	41	
	rEF (≤ 40%)	3 (9.7)	53 (100)	31	

A statistically significant association was found between the duration of T2DM, glycated hemoglobin, and ejection fraction (Table 3).

**Table 3:** Association of ejection fraction with different variables of study participants p-value, significance value; HbA1c, glycated hemoglobin; NT-proBNP, n-terminal pro-brain natriuretic peptide; pEF, preserved ejection fraction; mEF, Mid-range ejection fraction; rEF, reduced ejection fraction

Variables of Study Participants		Ejection Fraction n (%)				P-value
		pEF n = 236 (76.6)	mrEF n = 41 (13.3)	rEF n = 31 (10.1)	Total = 308	
Age Group (Years)	40–60	122 (79.7)	18 (11.8)	13 (8.5)	153	0.097
	61–80	111 (75.5)	20 (13.6)	16 (10.9)	147	
	≥80	3 (37.5)	3 (37.5)	2 (25.0)	8	
Duration of Type-2 Diabetes (Years)	10–20	220 (85.3)	22 (8.5)	16 (6.2)	258	< 0.001
	20–30	15 (32.6)	19 (41.3)	12 (26.1)	46	
	>30	1 (25.0)	0	3 (73)	4	
HbA1c	<6.5	23 (79.3)	6 (20.7)	0	29	0.039
	6.5–9.5	174 (76.3)	25 (11.0)	29 (12.7)	288	
	9.5	39 (76.5)	10 (19.6)	2 (3.9)	51	

There was a strong positive correlation (correlation coefficient = 0.712) between NT-proBNP and the duration of T2DM. Similarly, the ejection fraction of the participants showed a strong negative correlation (correlation coefficient = -0.743) with NT-proBNP. These correlations were statistically highly significant too (Table 4).

**Table 4:** Correlation of NT-proBNP with different variables of study participants T2DM, type-2 diabetes mellitus; NT-proBNP, n-terminal pro-brain natriuretic peptide ** Correlation is significant at the 0.01 level (two-tailed)

Variables	Correlation Coefficient	Sig. (Two-tailed)
Age	0.048	0.354
Duration of T2DM	0.712^**^	< 0.001
HbA1C	-0.063	0.215
Ejection Fraction %	-0.743^**^	< 0.001

**Figure 1:**
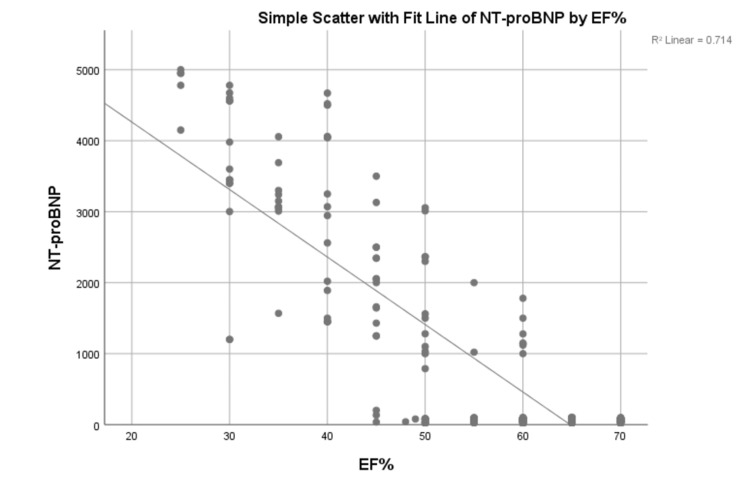
Scatter plot between EF and NT-proBNP NT-proBNP, n-terminal pro-brain natriuretic peptide; EF, ejection fraction

Figure 1 shows a simple scatter with a fit line of NT-proBNP by ejection fraction. It is evident that there is an inverse relationship between NT-proBNP level and ejection fraction.

## Discussion

The present hospital-based cross-sectional observational study aimed to assess the factors affecting NT-proBNP and EF for detecting potential heart failure in T2DM patients who do not exhibit overt heart failure symptoms. The study enrolled a total of 308 subjects with T2DM patients who satisfied the inclusion and exclusion criteria. Patients with the mentioned clinical conditions were excluded as these conditions have the potential to independently affect our study variables and outcome. The findings of this study may help in establishing the significance of early identification of the predictors of cardiac dysfunction in T2DM patients without clinical features of overt heart failure.

Studies conducted earlier have reported a high prevalence of cardiovascular complications in patients with T2DM. Research studies suggest that diabetic cardiomyopathy develops in patients with T2DM without the presence of typical cardiovascular risk factors like hypertension, dyslipidemia, or smoking. It has been observed that the risk of heart failure is higher in patients with T2DM than in nondiabetic individuals, even in the absence of clinical symptoms of heart failure. Moreover, elevated levels of NT-proBNP have been observed in T2DM patients, indicating an increased risk of cardiovascular complications, including heart failure [12]. Similarly, a reduction in ejection fraction has also been observed in T2DM patients, which is an important predictor of heart failure and complications [13]. Estimating NT-proBNP and left ventricular ejection fraction in T2DM patients is an important step in evaluating cardiac function, which helps in early identification, timely intervention, and management of cardiovascular complications effectively.

Elevated NT-proBNP was found more in the long duration of T2DM patients in this study. Additionally, the mean T2DM duration observed in this study was 17.1 ± 5.9 years. This finding coincides with a study conducted in India that reported a mean T2DM duration of 15.5 years [14]. The prolonged T2DM duration has been found associated with a higher risk of developing CVD in patients with T2DM [15]. Therefore, it is crucial to ensure optimal glycemic control and regular screening for CVD risk factors in patients with T2DM, especially those with longer T2DM duration. In this study, the majority of the study subjects (228, 74%) had HbA1c levels between 6.5-9.5%, which is higher than the recommended target. Similarly, suboptimal glycemic control was also found in patients with T2DM in studies conducted in the past [16,17]. Only a small proportion of the study subjects (29, 9.4%) had HbA1c levels below the recommended target of <6.5%, indicating that there is a need for better glycemic control awareness in this population. Good glycemic control has been shown to reduce the risk of microvascular and macrovascular complications in patients with diabetes [18]. Overall, these findings highlight the need for better glycemic control in patients with T2DM without clinical features of heart failure.

NT-proBNP is a sensitive biomarker for heart failure, and elevated NT-proBNP levels have been observed in patients with T2DM, even in the absence of clinical features of heart failure [19]. In this study, 98 (31.8%) participants had elevated NT-proBNP levels (> 125 pg/dl), indicating that a significant proportion of patients with T2DM may have underlying cardiac dysfunction even in the absence of overt heart failure symptoms. This observation is consistent with a previous study that has reported that patients with T2DM are at an increased risk of developing cardiovascular complications such as heart failure [20]. The relationship between BNP and age has also been well established, with NT-proBNP levels increasing with age [21], we also found a significant association between age and BNP level of study participants.

The statistically significant association (p < 0.001) found between T2DM duration, and an NT-proBNP level in this study indicates a possible relationship between the longer duration of T2DM and elevated NT-proBNP levels. This finding is consistent with previous studies that have reported an association between the duration of T2DM and elevated NT-proBNP levels [22,23]. Among the study subjects with normal NT-proBNP levels, 162 (71.1) had HbA1c levels between 6.5-9.5%, whereas among those with elevated NT-proBNP levels, 66 (28.9%) had HbA1c levels between 6.5-9.5%. This finding suggests that patients with elevated HbA1c levels are at a higher risk of developing elevated NT-proBNP, which is consistent with previous studies [24,25]. Hence, NT-proBNP levels can be used as a useful tool to identify patients at risk of developing cardiac dysfunction, which may prompt further evaluation and intervention to prevent overt heart failure.

The study found that the majority (236, 76.6%) of the subjects had preserved ejection fraction, while 41 (13.3%) had moderate ejection fraction, and 31 (130.1%) had reduced ejection fraction. Ejection fraction is an important measure of heart function, and a reduced ejection fraction is a marker of systolic dysfunction, which can increase the risk of heart failure and cardiovascular events. Finding low (moderate or reduced) ejection fraction in T2DM subjects without clinical features of heart failure is consistent with Rosno et al. [26].

In this study, preserved ejection fraction is the most common although the association between age and ejection fraction was found to be nonsignificant, it is possible that age-related changes in the myocardium could still be contributing to the observed differences in ejection fraction among the different age groups. This is consistent with a previous study that reported a high prevalence of preserved ejection fraction in patients with heart failure [27].

Furthermore, the association between T2DM duration and ejection fraction was found to be statistically significant, indicating that longer T2DM duration was associated with a higher probability of developing midrange and reduced ejection fraction. This finding is consistent with previous studies that have reported a significant association between T2DM duration and impaired left ventricular function [28]. It is believed that chronic hyperglycemia, insulin resistance, and oxidative stress are the key factors that contribute to the development of left ventricular dysfunction in patients with T2DM.

The current study found a significant association between the ejection fraction and HbA1c levels in diabetic patients highlighting the importance of glycemic control in preventing cardiac dysfunction in diabetic patients. Literature also shows a significant association between poor glycemic control and cardiac dysfunction in diabetes. Elevated HbA1c levels have been shown to cause structural and functional changes in the myocardium, such as increased collagen deposition and impaired cardiac energetics, which can lead to impaired cardiac function [29]. Elevated NT-proBNP levels are a marker of myocardial dysfunction and are strongly associated with the presence and severity of heart failure [30].

The current study has several limitations. First, the cross-sectional study design makes it difficult to establish a cause-effect relationship between T2DM duration and cardiac dysfunction. Secondly, this is a single-center study limiting the generalizability of the findings. Left ventricular global longitudinal strain and parameters of diastolic function were not studied in this project. Several other factors might work in conjunction, which could influence left ventricular function.

Further studies are needed to explore the factors contributing to poor glycemic control in this population and to develop effective interventions to improve glycemic control and reduce the risk of complications.

## Conclusions

Elevated NT-proBNP levels and impaired EF were found in a significant proportion of these patients, indicating an increased risk of cardiovascular complications. This study highlights a significant association between NT-proBNP and EF in patients with T2DM in those without overt heart failure symptoms. Furthermore, longer T2DM duration and higher HbA1c levels were found to be associated with elevated NT-proBNP levels. Conversely, longer T2DM duration and elevated BNP were linked to lower EF. These findings have important clinical implications as they suggest that monitoring NT-proBNP levels in patients with T2DM without clinical features of overt heart failure may help identify those at risk for decreased EF and potentially prevent heart failure. This study underscores the importance of regular cardiac screening and glycemic control in T2DM patients to prevent the development of heart failure and improve long-term outcomes. Further research is warranted to explore additional factors contributing to cardiac dysfunction in this population and to develop effective interventions for risk reduction.
